# Interindividual variability and its impact on the effectiveness of Janus kinase inhibitors in rheumatoid arthritis treatment

**DOI:** 10.3389/fmed.2025.1512501

**Published:** 2025-03-28

**Authors:** Cristina Martinez-Molina, Silvia Vidal, Cesar Diaz-Torne, Hye S. Park, Hèctor Corominas

**Affiliations:** ^1^Department of Pharmacy, Division of Medicines, Hospital Clínic de Barcelona, Barcelona, Spain; ^2^Department of Medicine, Universitat Autònoma de Barcelona (UAB), Bellaterra, Spain; ^3^Research Group of Inflammatory Diseases, Institut de Recerca Sant Pau (IR Sant Pau), Barcelona, Spain; ^4^Department of Rheumatology and Systemic Autoimmune Diseases, Hospital de la Santa Creu i Sant Pau, Barcelona, Spain; ^5^Research Group of Multi-Organ Damage and Rheumatology, Institut de Recerca Sant Pau (IR Sant Pau), Barcelona, Spain

**Keywords:** Janus kinase inhibitor, tofacitinib, baricitinib, upadacitinib, filgotinib, treatment effectiveness, rheumatoid arthritis

## Abstract

**Introduction:**

Achieving the primary treat-to-target (T2T) goal in rheumatoid arthritis (RA) remains challenging for many patients, reflecting limitations in the effectiveness of existing treatments. Our study examines factors influencing Janus kinase (JAK) inhibitor effectiveness by analyzing interindividual variability in demographic and clinical characteristics of real-world RA patients.

**Materials and methods:**

This observational retrospective study involves RA patients receiving tofacitinib, baricitinib, upadacitinib, or filgotinib between September 2017 and January 2025. Predictive factors of achieving the T2T goal at 6 months were identified through logistic regression analyses. Disparities in the treatment effectiveness retention based on predictive factors were assessed using the Kaplan–Meier estimate and compared with the log-rank test. The Cox model was applied to analyze whether the predictive factors identified could influence the retention of JAK inhibitor treatment effectiveness.

**Results:**

One hundred fifty patients were included: 81 (54%) achievers and 69 (46%) non-achievers of remission or, at least, low disease activity at 6 months of treatment. High disease activity at baseline, with respect to moderate activity, was identified as an unfavorable factor for achieving the T2T goal (Odds ratio adjusted: 0.96; 95% confidence interval: 0.92–0.99; *p* = 0.028). In treatment effectiveness retention rates, no differences were observed between patients with high versus moderate disease activity (*p* = 0.103). RA disease activity at baseline was not found to impact the survival of JAK inhibitor effectiveness (*p* = 0.106).

**Conclusion:**

In RA, high disease activity at the initiation of treatment with tofacitinib, baricitinib, upadacitinib, or filgotinib does not preclude an effective treatment response but is associated with an increased risk of therapeutic failure. Factors not related to the achievement of the T2T goal at 6 months of JAK inhibitor treatment include: age, female sex, body mass index, RA disease duration, seropositivity for rheumatoid factor, seropositivity for anti-cyclic citrullinated peptides, JAK inhibitor selectivity, type and number of prior biologic treatments, concomitant use and number of prior conventional synthetic disease-modifying antirheumatic drugs, and number of prior JAK inhibitors. These conclusions are derived from a retrospective real-world study and should be confirmed in prospective studies.

## 1 Introduction

Rheumatoid arthritis is a chronic, systemic, inflammatory, autoimmune disease characterized by progressive joint destruction and systemic manifestations (1–3). The pathogenesis of rheumatoid arthritis is largely attributed to the dysregulation of both innate and adaptive immune responses (1–3). In recent years, an advanced understanding of pro-inflammatory and anti-inflammatory effects of specific cytokines involved in the pathogenesis of rheumatoid arthritis has driven the development of targeted disease-modifying antirheumatic drugs (DMARDs), including biologic DMARDs (bDMARDs) and targeted synthetic DMARDs (tsDMARDs), i.e., Janus kinase (JAK) inhibitors.

JAK inhibitors, including tofacitinib, baricitinib, upadacitinib, and filgotinib, inhibit JAKs by competing with the biological substrate, the adenosine triphosphate (ATP) (4–6). The ATP concentration in patients with rheumatoid arthritis has been linked to the level of inflammation (7–9), which is related to rheumatoid arthritis disease activity. JAK inhibitors are indicated, after risk assessment, for the treatment of moderate to high active rheumatoid arthritis in adult patients who have shown an inadequate response to, or who are intolerant to one or more DMARDs (10–13), as well as being a treatment option for “difficult-to-treat” rheumatoid arthritis patients in real-world clinical practice (14–16).

Patients with rheumatoid arthritis receiving treatment with JAK inhibitors in real-world settings often differ from those typically included in randomized controlled trials (RCTs), where study populations are meticulously selected and rigorously monitored under predefined environments (17, 18). In clinical practice, real-world patients often exhibit interindividual differences, including diverse sociodemographic characteristics, rheumatoid arthritis disease activities, comorbidities, concomitant treatments, and treatment histories. This interindividual variability can contribute to the fact that not all eligible patients achieve the primary goal in rheumatoid arthritis treatment. According to the treat-to-target (T2T) recommendations (19, 20), which are supported by several rheumatoid arthritis guidelines (14, 21, 22), the defined primary goal is to achieve and maintain clinical remission or, at least, low disease activity.

The present study aims to evaluate patient- and drug-related factors to help understand interindividual variations in the treatment effectiveness of JAK inhibitors. Real-world evidence, derived from both prospective or retrospective studies, significantly complements the information obtained from RCTs, providing valuable insights that enhance healthcare decision-making (17, 18).

## 2 Materials and methods

### 2.1 Study design and patient population

This was an observational retrospective study involving real-world patients aged 18 years and older with rheumatoid arthritis, conducted at a tertiary-care university hospital in Spain. Rheumatoid arthritis was diagnosed based on the 2010 classification criteria, as outlined by both the American College of Rheumatology (ACR) and the European League Against Rheumatism (EULAR) (23). Rheumatoid arthritis patients who were treated with either tofacitinib, baricitinib, upadacitinib, or filgotinib between September 2017 and January 2025, and who had comprehensive data on treatment initiation, the first 6 months of treatment, treatment retention, and reasons for treatment discontinuation, were considered for inclusion in the study. Each included patient was individually informed about the study protocol and offered the option to decline participation. Clinical data were collected from electronic patient records.

### 2.2 Assessments

The primary outcome was the identification of predictive factors associated with achieving remission or low disease activity at the first 6 months of the JAK inhibitor treatment. The potential predictive factors taken into account were sociodemographic [age, sex, body mass index (BMI)], disease duration, disease activity at baseline [assessed through the Clinical Disease Activity Index (CDAI)], seropositivity [rheumatoid factor (RF) and anti-cyclic citrullinated peptide (anti-CCP) antibodies], JAK inhibitor selectivity (JAK-1 inhibition from lowest to highest: tofacitinib, baricitinib, upadacitinib, and filgotinib), number of prior rheumatoid arthritis treatments [previous conventional synthetic DMARDs (csDMARDs), previous bDMARDs, and previous JAK inhibitors], type of previous bDMARDs [tumor necrosis factor-alpha (TNF-*α*) inhibitor, interleukin 6 (IL6) inhibitor, abatacept, rituximab, and anakinra], and concomitant rheumatoid arthritis treatments (presence or absence of csDMARDs). The first 6 months of the JAK inhibitor treatment were selected to assess the primary outcome, in line with the ACR-EULAR recommendations (14, 21, 22) suggesting treatment modification if the T2T goal is not achieved at this time period. Disease activity was measured using the CDAI scale, as recommended to ensure unbiased outcomes in comparative effectiveness clinical studies involving drugs such as JAK inhibitors, which have a significant impact on acute phase reactants (24). The CDAI was classified according to the latest ACR recommendations (25), into remission (CDAI ≤2.8), low disease activity (CDAI ≤ 10), moderate disease activity (CDAI ≤ 22), and high disease activity (CDAI > 22).

The secondary outcome was to determine whether the predictive factors identified linked to the achievement of the T2T goal at the first 6 months, also contribute to differences in the long-term effectiveness of the JAK inhibitor treatment. Long-term effectiveness was evaluated through the retention of the JAK inhibitor treatment, with treatment discontinuation due to lack of effectiveness considered as the event of interest. The retention of treatment captured the time interval from treatment initiation to definitive treatment discontinuation.

### 2.3 Statistical analyses

Demographic and clinical patient characteristics were separately described in relation to the achievement of the T2T goal at the first 6 months of the JAK inhibitor treatment. Differences between T2T goal achievers and non-achievers were assessed using the Mann–Whitney test for ordinal or quantitative variables, and the Fisher’s exact test for categorical variables. Ordinal and quantitative variables are presented as medians and interquartile ranges (IQR), while categorical variables are described using absolute numbers (*n*) and percentages (%).

Predictive factors of achieving the T2T goal at the first 6 months of the JAK inhibitor treatment were identified through logistic regression analyses (bivariate and multivariate). The covariables with a *p* < 0.1 in the bivariate analysis were included in the multivariate model.

The retention of the JAK inhibitor treatment was evaluated using both the Kaplan–Meier estimate and the Cox proportional hazard regression model. The Kaplan–Meier estimate was employed to analyze survival curves for the discontinuation reason of lack of treatment effectiveness, comparing patients with moderate and high disease activity at baseline using the log-rank test. The bivariate Cox model was applied to analyze whether rheumatoid arthritis disease activity at baseline could influence the retention of the JAK inhibitor treatment.

All statistical analyses were conducted using Stata software version 12. Statistical significance was defined at a *p* < 0.05.

### 2.4 Ethics approval and consent to participate

Approval was granted by the ethics committee of a hospital (IIBSP-JAG-2023-168). This study involving human participants was conducted in accordance with the Declaration of Helsinki.

## 3 Results

One hundred fifty rheumatoid arthritis patients treated with a JAK inhibitor were included in this study. Table 1 provides a summary of their demographic and clinical characteristics.

**Table 1:** Demographic and clinical patient characteristics.

Parameter	T2T achievers at 6 months (*n* = 81)	T2T non-achievers at 6 months (*n* = 69)	*p*-value
Age (years), median [IQR]	66 [56–71]	64 [47–69]	0.071
Sex (female), n (%)	67 (82.7)	61 (88.4)	0.363
BMI (weight/height^2^), median [IQR]	27.0 [24.9–29.0]	27.0 [22.9–31.1]	0.903
RA disease duration (years), median [IQR]	14.0 [5.0–24.0]	13.0 [6.0–19.0]	0.461
RA seropositivity, n (%)			
RF	53 (65.4)	39 (56.5)	0.314
Anti-CCP	65 (80.3)	48 (69.6)	0.093
JAK inhibitor type, n (%)			0.366
Tofacitinib	24 (29.6)	27 (39.1)	
Baricitinib	46 (56.8)	30 (43.5)	
Upadacitinib	6 (7.4)	5 (7.3)	
Filgotinib	5 (6.2)	7 (10.1)	
Concomitant csDMARD use, n (%)	24 (29.6)	19 (27.5)	0.857
Methotrexate	19 (23.5)	10 (14.5)	0.214
Leflunomide	2 (2.5)	1 (1.5)	1.000
Sulfasalazine	0 (0.0)	5 (7.3)	0.019
Hydroxychloroquine	4 (4.9)	5 (7.3)	0.733
Concomitant GC use, n (%)	38 (46.9)	55 (79.7)	<0.001
PDN dose (mg/day), median [IQR]	0.0 [0.0–5.0]	5.0 [0.0–7.5]	<0.001
RA disease activity, median [IQR]			
CDAI at baseline	22.0 [16.0–28.0]	28.0 [21.0–34.0]	<0.001
CDAI at 6 months	5.0 [3.0–7.0]	23.0 [15.9–31.0]	<0.001
Previous csDMARDs, n (%)			
Methotrexate	74 (91.4)	55 (79.7)	0.058
Leflunomide	34 (42.0)	36 (52.2)	0.139
Sulfasalazine	19 (23.5)	18 (26.1)	0.849
Hydroxychloroquine	23 (28.4)	15 (21.7)	0.452
Gold salts	11 (13.6)	4 (5.8)	0.171
Chloroquine	10 (12.4)	2 (2.9)	0.038
Mycophenolate	5 (6.2)	1 (1.5)	0.218
Azathioprine	3 (3.7)	2 (2.9)	1.000
Cyclophosphamide	6 (7.4)	1 (1.5)	0.125
Cyclosporine	3 (3.7)	2 (2.9)	1.000
Penicillamine	2 (2.5)	0 (0.0)	0.500
Previous bDMARDs, n (%)			
Adalimumab	32 (39.5)	41 (59.4)	0.021
Certolizumab	19 (23.5)	29 (42.0)	0.022
Etanercept	23 (28.4)	38 (55.1)	0.001
Golimumab	15 (18.5)	16 (23.2)	0.546
Infliximab	12 (14.8)	11 (15.9)	1.000
Tocilizumab	33 (40.7)	39 (56.5)	0.071
Sarilumab	12 (14.8)	16 (23.2)	0.212
Abatacept	28 (34.6)	35 (50.7)	0.049
Rituximab	16 (19.8)	18 (26.1)	0.435
Anakinra	0 (0.0)	4 (5.8)	0.043
Previous JAK inhibitors, n (%)			
Tofacitinib	13 (16.1)	18 (26.1)	0.158
Baricitinib	10 (12.4)	17 (24.6)	0.058
Upadacitinib	1 (1.2)	2 (2.9)	0.594
Filgotinib	0 (0.0)	1 (1.5)	0.460

T2T, treat-to-target; IQR, interquartile range [P25–P75]; BMI, body mass index (kg/m^2^); RA, rheumatoid arthritis; RF, rheumatoid factor; anti-CCP, anti-cyclic citrullinated peptide; JAK, Janus Kinase; csDMARD, conventional synthetic Disease Modifying Anti-Rheumatic Drug; GC, glucocorticoid; PDN, prednisone; CDAI, Clinical Disease Activity Index; bDMARD, biologic Disease Modifying Anti-Rheumatic Drug.

Similar distribution of JAK inhibitor type was observed between patients achieving and not achieving the T2T goal at 6 months of treatment. At JAK inhibitor treatment initiation, T2T goal achievers and non-achievers presented comparable years of age, sex distribution, BMI, years of rheumatoid arthritis disease duration, seropositivity considering RF and anti-CCP antibodies, prior csDMARD use, and prior JAK inhibitor use.

In terms of prior bDMARD use, patients who did not achieve the T2T goal at 6 months of the JAK inhibitor treatment, exhibited a higher discontinuation of prior treatment with adalimumab (*p =* 0.021), certolizumab (*p =* 0.022), etanercept (*p =* 0.001), abatacept (*p =* 0.049), and anakinra (*p =* 0.043). No significant differences were noted between the T2T goal achievers and non-achievers regarding the discontinuation of prior treatment with golimumab, infliximab, tocilizumab, sarilumab, and rituximab.

Concerning rheumatoid arthritis disease activity, patients who did not achieve the T2T goal presented higher disease activity both at baseline (*p* < 0.001) and at 6 months of the JAK inhibitor treatment (*p* < 0.001).

Regarding concomitant rheumatoid arthritis treatment, patients who did not achieve the T2T goal presented a higher use of concomitant glucocorticoids (*p* < 0.001) and received higher equivalent doses of prednisone (*p* < 0.001) compared to those who did achieve the T2T goal at the first 6 months of the JAK inhibitor treatment. About concomitant csDMARD use, sulfasalazine use was found to be higher among T2T goal non-achievers compared to achievers, whereas the concomitant use of methotrexate, leflunomide, and hydroxychloroquine was comparable between both groups of patients.

The results from the logistic regression analyses are presented in Table 2.

**Table 2:** Predictive factors for achieving the T2T goal at 6 months of JAK inhibitor treatment.

Covariate	Bivariate analysis		Multivariate analysis	
	OR [95% CI]	*p*-value	ORadj [95% CI]	*p*-value
RA disease activity at baseline (HDA)	0.94 [0.91–0.98]	0.001	0.96 [0.92–0.99]	0.028
Previous bDMARDs (number)	0.79 [0.68–0.91]	0.001	0.77 [0.37–1.61]	0.494
Prior TNF-α inhibitor (use)	0.67 [0.52–0.87]	0.002	1.06 [0.46–2.43]	0.899
Prior IL6 inhibitor (use)	0.59 [0.36–0.96]	0.032	1.29 [0.45–3.67]	0.630
Age (years)	1.03 [1.00–1.05]	0.043	1.03 [1.00–1.06]	0.072
Previous JAK inhibitors (number)	0.61 [0.38–0.99]	0.046	0.68 [0.38–1.20]	0.180
Prior abatacept (use)	0.51 [0.27–0.99]	0.047	1.20 [0.36–3.94]	0.769
Anti-CCP (seropositivity)	1.78 [0.84–3.76]	0.133		
Previous csDMARDs (number)	1.17 [0.93–1.46]	0.181		
Prior anakinra (use)	1.01 [0.90–1.73]	0.186		
RF (seropositivity)	1.46 [0.75–2.82]	0.265		
RA disease duration (years)	1.02 [0.99–1.05]	0.302		
Sex (female)	0.63 [0.25–1.60]	0.329		
Prior rituximab (use)	0.70 [0.32–1.50]	0.357		
BMI (weight/height^2^)	0.98 [0.92–1.05]	0.618		
Concomitant csDMARD (use)	1.11 [0.54–2.26]	0.778		
JAK inhibitor (JAK-1 selectivity)	1.02 [0.70–1.45]	0.902		

OR, Odds Ratio; CI, Confidence Interval; ORadj, Odds Ratio adjusted; RA, rheumatoid arthritis; HDA, high disease activity; bDMARD, biologic Disease Modifying Anti-Rheumatic Drug; TNF-α, tumor necrosis factor-alpha; IL6, interleukin 6; JAK, Janus Kinase; anti-CCP, anti-cyclic citrullinated peptide; csDMARD, conventional synthetic Disease Modifying Anti-Rheumatic Drug; RF, rheumatoid factor; BMI, body mass index (kg/m^2^).

High disease activity at baseline, with respect to moderate activity, was identified as a potential unfavorable factor for achieving the T2T goal at the first 6 months of the JAK inhibitor treatment [unadjusted odds ratio (OR): 0.94; 95% confidence interval (CI): 0.91–0.98; *p* = 0.001]. Similarly, the prior number of JAK inhibitor treatments (OR: 0.61; 95% CI: 0.38–0.99; *p* = 0.046), the prior number of bDMARD treatments (OR: 0.79; 95% CI: 0.68–0.91; *p* = 0.001), the prior use of a TNF-*α* inhibitor (OR: 0.67; 95% CI: 0.52–0.87; *p* = 0.002), the prior use of a IL6 inhibitor (OR: 0.59; 95% CI: 0.36–0.96; *p* = 0.032), and the prior use of abatacept (OR: 0.51; 95% CI: 0.27–0.99; *p* = 0.047) were considered as potential unfavorable factors, while age was determined as a potential favorable factor (OR: 1.03; 95% CI: 1.00–1.05; *p* = 0.043).

Through multivariate model, high disease activity at baseline was established as an unfavorable factor for achieving the T2T goal at the first 6 months of the JAK inhibitor treatment, with an adjusted odds ratio (ORadj) of 0.96 (95% CI: 0.92–0.99; *p* = 0.028). None of the following factors were identified as predictive, either favorable or unfavorable: age; female sex; BMI; rheumatoid arthritis disease duration; seropositivity for rheumatoid factor or anti-CCP; JAK inhibitor selectivity; concomitant use of csDMARDs; the number of csDMARDs, bDMARDs, or JAK inhibitors previously used; or prior use of a TNF-*α* inhibitor, a IL6 inhibitor, abatacept, rituximab, or anakinra.

**Figure 1:**
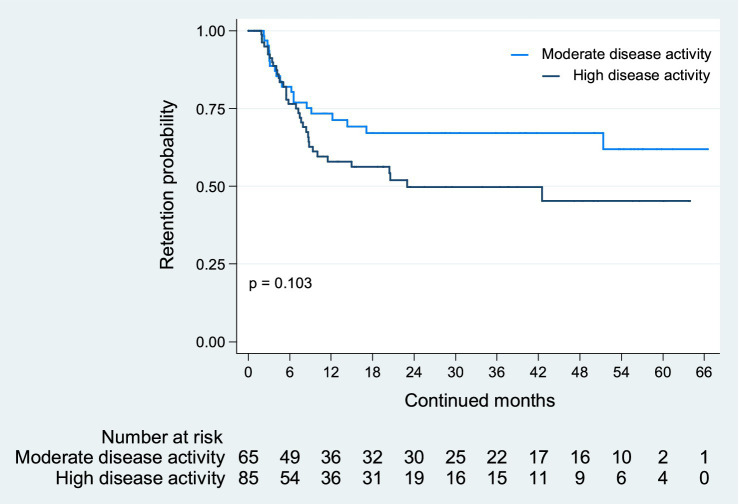
JAK inhibitor retention due to lack of treatment effectiveness. The retention of JAK inhibitors due to lack of treatment effectiveness was comparable between patients with moderate and high disease activity at baseline (*p* = 0.103).

The retention of JAK inhibitor treatment, due to discontinuation for lack of treatment effectiveness, is depicted in Figure 1.

There were no observed differences in treatment retention rates between patients with high versus moderate disease activity at baseline (*p* = 0.103). Rheumatoid arthritis disease activity at baseline was not found to impact the survival of the JAK inhibitor treatment effectiveness [hazard ratio (HR) for patients with high disease activity at baseline: 1.58; 95% CI: 0.91–2.74; *p* = 0.106].

## 4 Discussion

By considering the interindividual variability in the demographic and clinical characteristics of real-world patients, this research assessed both patient- and drug-related factors that may influence the effectiveness of JAK inhibitors in rheumatoid arthritis treatment.

Among factors associated with the effectiveness of JAK inhibitors, baseline rheumatoid arthritis disease activity has been identified. Logistic regression analyses have determined it to be a predictive factor for resistance to achieving remission or low disease activity at the first 6 months of treatment (ORadj for high disease activity: 0.96; 95% CI: 0.92–0.99; *p* = 0.028). These findings align with those of Hayashi et al. (26), who linked baseline rheumatoid arthritis disease activity to resistance in achieving low disease activity in patients treated with tofacitinib or baricitinib. According to the Cheng-Prusoff equation, high intracellular ATP concentrations—which correspond to a higher degree of inflammation or rheumatoid arthritis disease activity (7–9)—displace competitive inhibitors, such as tofacitinib, baricitinib, upadacitinib, or filgotinib, from binding to their target, thereby influencing the clinical response to treatment (27–29). However, it is important to note that our long-term analysis, assessed through treatment retention, points out that high disease activity does not preclude an effective response to these small molecules in rheumatoid arthritis treatment (HR for patients with high baseline disease activity: 1.58; 95% CI: 0.91–2.74; *p* = 0.106). Our findings lead us to suggest that tailoring the dosing regimen of JAK inhibitors according to the level of rheumatoid arthritis disease activity might enhance treatment effectiveness. In this context, Takeuchi et al. (30) demonstrated that reducing the dose of baricitinib in patients with sustained disease activity effectively maintains disease control, a finding now included as a recommendation in the drug’s product information (11).

With regard to age, Fleischmann et al. (31) found that the effectiveness of JAK inhibitors was comparable between younger and older rheumatoid arthritis patients. Furthermore, indirect studies have not identified age as a factor associated with the discontinuation of these small molecules due to lack of effectiveness (16, 32). Consistent with these findings, our results suggest that age does not influence the effectiveness of tofacitinib, baricitinib, upadacitinib, or filgotinib treatments in rheumatoid arthritis. It is worth mentioning that the age of our patients ranges between 64 years in subjects who did not achieve the T2T goal at 6 months of JAK inhibitor treatment and 66 years in T2T achievers.

About sex, prior research has shown no significant differences in the effectiveness of JAK inhibitors between female and male patients (16, 32–34). Martinez-Molina et al. (34) highlighted the importance of employing a comprehensive set of validated composite indices to assess rheumatoid arthritis disease activity. This recommendation is particularly relevant given the potential misclassification of disease activity in female patients when using the 28-joint Disease Activity Score (DAS28) with erythrocyte sedimentation rate (ESR), which may be influenced by sex-related differences in ESR levels (34–37). In line with these insights, the present study did not identify female sex as a factor affecting the effectiveness of JAK inhibitors in the treatment of rheumatoid arthritis.

In reference to BMI, the current evidence suggests that BMI does not impact the treatment effectiveness of these tsDMARDs in adult patients with rheumatoid arthritis (38, 39). This observation is consistent with the findings of our study.

Another consideration is the rheumatoid arthritis disease duration. Previous evidence has suggested that it is not linked to JAK inhibitor treatment effectiveness (16, 32, 33). Similarly, our study found no connection between disease duration and the effectiveness of tofacitinib, baricitinib, upadacitinib, or filgotinib treatments.

Concerning rheumatoid arthritis seropositivity, published studies indicate that neither RF nor anti-CCP positivity influences the treatment effectiveness of these small molecules (16, 33, 40). RF and/or anti-CCP positivity has been identified in several studies to be associated with enhanced treatment effectiveness of rituximab, abatacept, and TNF-*α* inhibitors in patients with rheumatoid arthritis (41–46). The largest study, a registry involving 2,583 patients, identified a positive association between seropositivity and treatment effectiveness to rituximab and abatacept, but found no association with TNF-α inhibitors (45). Consistent with previous evidence, regarding JAK inhibitors, the present study did not find rheumatoid arthritis seropositivity to be associated with treatment effectiveness.

As for JAK inhibitor selectivity, there is currently no conclusive answer due to the lack of head-to-head randomized clinical trials comparing JAK inhibitors (47). However, Traves et al. (48) conducted a study that integrated *in vitro* and clinical pharmacokinetics to differentiate the inhibition and selectivity of tofacitinib, baricitinib, upadacitinib, and filgotinib within the context of rheumatoid arthritis. These authors concluded that the four tsDMARDs exhibit similar effectiveness, which can be attributed to their comparable inhibition of JAK-1 signaling (48). The evidence supports the notion that in our study, no differences were observed in the effectiveness of these small molecules with respect to JAK-1 selectivity in rheumatoid arthritis treatment.

Other important factors to consider include the number of previous csDMARD treatments and the concomitant use of csDMARDs. Both JAK inhibitors and csDMARDs are used in the treatment of rheumatoid arthritis, each targeting distinct immune pathways through different mechanisms of action. To date, there is a lack of strong evidence directly comparing JAK inhibitor monotherapy (49–54) with the concomitant use of csDMARDs (51, 54–66). The EULAR Task Force recommends maintaining csDMARDs when initiating treatment with a JAK inhibitor (14). According to their guidelines, the csDMARD dose can be reduced to mitigate the risk of adverse events (14). Consistent with our findings, neither the number of previous csDMARD treatments nor the concomitant use of csDMARDs seems to determine the effectiveness of JAK inhibitors in the treatment of rheumatoid arthritis.

In terms of the number of previous bDMARDs treatments, Hayashi et al. (26) identified this factor as a predictor of resistance to achieving low disease activity with baricitinib, but not with tofacitinib. Other studies have not recognized it as a factor associated with the effectiveness of JAK inhibitor treatment (16, 33). The immune dysregulation in rheumatoid arthritis may involve a complex interplay among various cytokines, suggesting that blocking a single cytokine does not necessarily lead to the achievement of the T2T goal. In this context, it may be necessarily to target another specific molecule or even an entire pathway. The findings of the present study indicate that neither the number of previous bDMARD treatments nor the specific mechanism of action of a prior bDMARD significantly impacts the treatment effectiveness of tofacitinib, baricitinib, upadacitinib, or filgotinib.

Regarding the number of previous JAK inhibitor treatments, both the JAK inhibitor cycling strategy and several studies suggest that this factor does not influence the effectiveness of subsequent small molecule treatments (16, 32, 67). The different chemical structure of each JAK inhibitor provides a specific selective inhibition profile toward its target, which likely accounts for the variability in clinical responses observed in real-world practice. As recommended by the EULAR Task Force, if treatment with a JAK inhibitor fails, a change in treatment to another tsDMARD or bDMARD should be considered, given the lack of reliable predictors of treatment response in individual patients (14).

This study has several limitations that may affect the interpretation of the results. Firstly, its observational retrospective nature means that the findings should be viewed as hypothesis-generating and confirmed in prospective studies. Secondly, the population size and the fact that the study was conducted at a single centre should be considered when extrapolating the results to the broader population. Thirdly, our study did not include small molecules other than tofacitinib, baricitinib, upadacitinib, or filgotinib —the JAK inhibitors presently approved in Europe for rheumatoid arthritis treatment— such as peficitinib. Fourthly, adjustments in administration frequency or in dosage of JAK inhibitor treatment were not recorded; it was presupposed that all patients received their required administration frequency and dosage as per the approved guidelines. Fifthly, the imbalance in sex distribution, which mirrors real-world clinical data of a disease that primarily affects females more than males. Sixthly, patient comorbidities were not considered; it was assumed that the JAK inhibitor treatment was prescribed in accordance with the recommendations of the approved guidelines. Nonetheless, the findings are consistent with existing evidence.

The main strength of our study is its identification of factors influencing the effectiveness of JAK inhibitors in rheumatoid arthritis treatment, achieved by examining the interindividual variability in the demographic and clinical characteristics of real-world patients. This is particularly significant as it provides insights that can complement those derived from RCTs.

In summary, the results of our retrospective real-world study suggest that in rheumatoid arthritis, high disease activity at the initiation of treatment with tofacitinib, baricitinib, upadacitinib, or filgotinib does not preclude an effective treatment response but is associated with an increased risk of therapeutic failure. This leads us to suggest that tailoring the dosing regimen of JAK inhibitors according to the level of rheumatoid arthritis disease activity might enhance treatment effectiveness. By contrast, the following factors were not related to JAK inhibitor effectiveness in rheumatoid arthritis treatment: age, female sex, BMI, disease duration, seropositivity, JAK inhibitor selectivity, type and number of prior bDMARDs, concomitant use and number of prior csDMARDs, and number of prior JAK inhibitors. These findings can substantially enhance the insights obtained from randomized controlled trials, offering critical information that can improve healthcare decision-making.

## Data Availability

The raw data supporting the conclusions of this article will be made available by the authors, without undue reservation.

## References

[1] Scott DL Wolfe F Huizinga TW. Rheumatoid arthritis. Lancet. (2010) 376:1094–108. doi: 10.1016/S0140-6736(10)60826-4, PMID: 20870100

[2] McInnes IB Schett G. The pathogenesis of rheumatoid arthritis. N Engl J Med. (2011) 365:2205–19. doi: 10.1056/NEJMra1004965, PMID: 22150039

[3] Smolen JS Aletaha D McInnes IB. Rheumatoid arthritis. Lancet. (2016) 388:2023–38. doi: 10.1016/S0140-6736(16)30173-8, PMID: 27156434

[4] Lin CM Cooles FA Isaacs JD. Basic mechanisms of JAK inhibition. Mediterr J Rheumatol. (2020) 31:100–4. doi: 10.31138/mjr.31.1.100, PMID: 32676567 PMC7361186

[5] Nash P Kerschbaumer A Dörner T Dougados M Fleischmann RM Geissler K. Points to consider for the treatment of immune-mediated inflammatory diseases with Janus kinase inhibitors: a consensus statement. Ann Rheum Dis. (2021) 80:71–87. doi: 10.1136/annrheumdis-2020-218398, PMID: 33158881 PMC7788060

[6] Shawky AM Almalki FA Abdalla AN Abdelazeem AH Gouda AM. A comprehensive overview of globally approved JAK inhibitors. Pharmaceutics. (2022) 14:1001. doi: 10.3390/pharmaceutics14051001, PMID: 35631587 PMC9146299

[7] Tripathy A Padhan P Swain N Raghav SK Gupta B. Increased extracellular ATP in plasma of rheumatoid arthritis patients activates CD8+T cells. Arch Med Res. (2021) 52:423–33. doi: 10.1016/j.arcmed.2020.12.010, PMID: 33541740

[8] da Silva JLG Passos DF Bernardes VM Leal DBR. ATP and adenosine: role in the immunopathogenesis of rheumatoid arthritis. Immunol Lett. (2019) 214:55–64. doi: 10.1016/j.imlet.2019.08.009, PMID: 31479688

[9] Baroja-Mazo A Pelegrín P. Modulating P2X7 receptor signaling during rheumatoid arthritis: new therapeutic approaches for bisphosphonates. J Osteoporos. (2012) 2012:408242:1–7. doi: 10.1155/2012/408242, PMID: 22830074 PMC3399340

[10] Xeljanz (Tofacitinib): European Public Assessment Report (EPAR). (2024). European medicines agency (EMA), the Netherlands. Available online at: https://www.ema.europa.eu/en/medicines/human/EPAR/xeljanz (Accessed August 16, 2024).

[11] Olumiant (Baricitinib): European Public Assessment Report (EPAR). (2024). European medicines agency (EMA), the Netherlands. Available online at: https://www.ema.europa.eu/en/medicines/human/EPAR/olumiant (Accessed August 16, 2024).

[12] Rinvoq (Upadacitinib): European Public Assessment Report (EPAR). (2024). European medicines agency (EMA), the Netherlands. Available online at: https://www.ema.europa.eu/en/medicines/human/EPAR/rinvoq (Accessed August 16, 2024).

[13] Jyseleca (Filgotinib): European Public Assessment Report (EPAR). (2024). European medicines agency (EMA), the Netherlands. Available online at: https://www.ema.europa.eu/en/medicines/human/EPAR/jyseleca (Accessed August 16, 2024).

[14] Smolen JS Landewé RBM Bergstra SA Kerschbaumer A Sepriano A Aletaha D. EULAR recommendations for the management of rheumatoid arthritis with synthetic and biological disease-modifying antirheumatic drugs: 2022 update. Ann Rheum Dis. (2023) 82:3–18. doi: 10.1136/ard-2022-223356, PMID: 36357155

[15] Nagy G Roodenrijs NMT Welsing PM Kedves M Hamar A van der Goes MC. EULAR definition of difficult-to-treat rheumatoid arthritis. Ann Rheum Dis. (2021) 80:31–5. doi: 10.1136/annrheumdis-2020-217344, PMID: 33004335 PMC7788062

[16] Martinez-Molina C Gich I Diaz-Torné C Park HS Feliu A Vidal S. Patient-related factors influencing the effectiveness and safety of Janus kinase inhibitors in rheumatoid arthritis: a real-world study. Sci Rep. (2024) 14:172. doi: 10.1038/s41598-023-50379-8, PMID: 38168532 PMC10761698

[17] Mahajan R. Real world data: additional source for making clinical decisions. Int J Appl Basic Med Res. (2015) 5:82. doi: 10.4103/2229-516X.157148, PMID: 26097811 PMC4456898

[18] Sherman RE Anderson SA Dal Pan GJ Gray GW Gross T Hunter NL. Real-world evidence - what is it and what can it tell us? N Engl J Med. (2016) 375:2293–7. doi: 10.1056/NEJMsb1609216, PMID: 27959688

[19] Smolen JS Aletaha D Bijlsma JW Breedveld FC Boumpas D Burmester G. Treating rheumatoid arthritis to target: recommendations of an international task force. Ann Rheum Dis. (2010) 69:631–7. doi: 10.1136/ard.2009.123919, PMID: 20215140 PMC3015099

[20] Smolen JS Breedveld FC Burmester GR Bykerk V Dougados M Emery P. Treating rheumatoid arthritis to target: 2014 update of the recommendations of an international task force. Ann Rheum Dis. (2016) 75:3–15. doi: 10.1136/annrheumdis-2015-207524, PMID: 25969430 PMC4717393

[21] Fraenkel L Bathon JM England BR St Clair EW Arayssi T Carandang K. American College of Rheumatology guideline for the treatment of rheumatoid arthritis. Arthritis Rheumatol. (2021) 73:924–39. doi: 10.1002/acr.24596, PMID: 34101387 PMC9273041

[22] Singh JA Furst DE Bharat A Curtis JR Kavanaugh AF Kremer JM. Update of the 2008 American College of Rheumatology recommendations for the use of disease-modifying antirheumatic drugs and biologic agents in the treatment of rheumatoid arthritis. Arthritis Care Res. (2012) 64:625–39. doi: 10.1002/acr.21641, PMID: 22473917 PMC4081542

[23] Aletaha D Neogi T Silman AJ Funovits J Felson DT Bingham CO 3rd. 2010 rheumatoid arthritis classification criteria: an American College of Rheumatology/European league against rheumatism collaborative initiative. Ann Rheum Dis. (2010) 69:1580–8. doi: 10.1136/ard.2010.138461, PMID: 20699241

[24] Janke K Kiefer C McGauran N Richter B Krause D Wieseler B. A systematic comparison of different composite measures (DAS 28, CDAI, SDAI, and Boolean approach) for determining treatment effects on low disease activity and remission in rheumatoid arthritis. BMC Rheumatol. (2022) 6:82. doi: 10.1186/s41927-022-00314-7, PMID: 36482451 PMC9732992

[25] England BR Tiong BK Bergman MJ Curtis JR Kazi S Mikuls TR. Update of the American college of rheumatology recommended rheumatoid arthritis disease activity measures. Arthritis Care Res. (2019) 71:1540–55. doi: 10.1002/acr.24042, PMID: 31709779 PMC6884664

[26] Hayashi S Tachibana S Maeda T Yamashita M Shirasugi I Yamamoto Y. Real-world comparative study of the efficacy of Janus kinase inhibitors in patients with rheumatoid arthritis: the ANSWER cohort study. Rheumatology. (2023) 63:3033–41. doi: 10.1093/rheumatology/kead543, PMID: 37924201

[27] Knight ZA Shokat KM. Features of selective kinase inhibitors. Chem Biol. (2005) 12:621–37. doi: 10.1016/j.chembiol.2005.04.011, PMID: 15975507

[28] Smyth LA Collins I. Measuring and interpreting the selectivity of protein kinase inhibitors. J Chem Biol. (2009) 2:131–51. doi: 10.1007/s12154-009-0023-9, PMID: 19568781 PMC2725273

[29] Thorarensen A Banker ME Fensome A Telliez JB Juba B Vincent F. ATP-mediated kinome selectivity: the missing link in understanding the contribution of individual JAK kinase isoforms to cellular signaling. ACS Chem Biol. (2014) 9:1552–8. doi: 10.1021/cb5002125, PMID: 24814050

[30] Takeuchi T Genovese MC Haraoui B Li Z Xie L Klar R. Dose reduction of baricitinib in patients with rheumatoid arthritis achieving sustained disease control: results of a prospective study. Ann Rheum Dis. (2019) 78:171–8. doi: 10.1136/annrheumdis-2018-213271, PMID: 30194275 PMC6352419

[31] Fleischmann R Alam J Arora V Bradley J Schlichting DE Muram D. Safety and efficacy of baricitinib in elderly patients with rheumatoid arthritis. RMD Open. (2017) 3:e000546. doi: 10.1136/rmdopen-2017-000546, PMID: 29071120 PMC5640108

[32] Ebina K Hirano T Maeda Y Yamamoto W Hashimoto M Murata K. Factors affecting drug retention of Janus kinase inhibitors in patients with rheumatoid arthritis: the ANSWER cohort study. Sci Rep. (2022) 12:134. doi: 10.1038/s41598-021-04075-0, PMID: 34997059 PMC8742057

[33] Pombo-Suarez M Sanchez-Piedra C Gómez-Reino J Lauper K Mongin D Iannone F. After JAK inhibitor failure: to cycle or to switch, that is the question -data from the JAK-pot collaboration of registries. Ann Rheum Dis. (2023) 82:175–81. doi: 10.1136/ard-2022-222835, PMID: 36100351

[34] Martinez-Molina C Feliu A Park HS Juanes A Diaz-Torne C Vidal S. Are there sex-related differences in the effectiveness of Janus kinase inhibitors in rheumatoid arthritis patients? J Clin Med. (2024) 13:2355. doi: 10.3390/jcm13082355, PMID: 38673626 PMC11050893

[35] Miller A Green M Robinson D. Simple rule for calculating normal erythrocyte sedimentation rate. Br Med J. (1983) 286:266. doi: 10.1136/bmj.286.6361.266 PMC1546487 6402065

[36] Alende-Castro V Alonso-Sampedro M Vazquez-Temprano N Tuñez C Rey D García-Iglesias C. Factors influencing erythrocyte sedimentation rate in adults: new evidence for an old test. Medicine. (2019) 98:e16816. doi: 10.1097/MD.0000000000016816, PMID: 31441853 PMC6716712

[37] Sokka T Toloza S Cutolo M Kautiainen H Makinen H Gogus F. QUEST-RA group. Women, men, and rheumatoid arthritis: analyses of disease activity, disease characteristics, and treatments in the QUEST-RA study. Arthritis Res Ther. (2009) 11:R7. doi: 10.1186/ar2591, PMID: 19144159 PMC2688237

[38] Dikranian AH Gonzalez-Gay MA Wellborne F Álvaro-Gracia JM Takiya L Stockert L. Efficacy of tofacitinib in patients with rheumatoid arthritis stratified by baseline body mass index: an analysis of pooled data from phase 3 studies. RMD Open. (2022) 8:e002103. doi: 10.1136/rmdopen-2021-002103, PMID: 35577477 PMC9114845

[39] Balsa A Wassenberg S Tanaka Y Tournadre A Orzechowski HD Rajendran V. Effect of Filgotinib on body mass index (BMI) and effect of baseline BMI on the efficacy and safety of Filgotinib in rheumatoid arthritis. Rheumatol Ther. (2023) 10:1555–74. doi: 10.1007/s40744-023-00599-1, PMID: 37747626 PMC10654312

[40] Bird P Hall S Nash P Connell CA Kwok K Witcombe D. Treatment outcomes in patients with seropositive versus seronegative rheumatoid arthritis in phase III randomised clinical trials of tofacitinib. RMD Open. (2019) 5:e000742. doi: 10.1136/rmdopen-2018-000742, PMID: 30886732 PMC6397430

[41] Narvaez J Díaz-Torné C Ruiz JM Hernandez MV Torrente-Segarra V Ros S. Predictors of response to rituximab in patients with active rheumatoid arthritis and inadequate response to anti-TNF agents or traditional DMARDs. Clin Exp Rheumatol. (2011) 29:991–7. PMID: 22133052

[42] Isaacs JD Cohen SB Emery P Tak PP Wang J Lei G. Effect of baseline rheumatoid factor and anticitrullinated peptide antibody serotype on rituximab clinical response: a meta-analysis. Ann Rheum Dis. (2013) 72:329–36. doi: 10.1136/annrheumdis-2011-201117, PMID: 22689315

[43] Daïen CI Morel J. Predictive factors of response to biological disease modifying antirheumatic drugs: towards personalized medicine. Mediat Inflamm. (2014) 2014:386148. doi: 10.1155/2014/386148, PMID: 24523570 PMC3913459

[44] Gardette A Ottaviani S Tubach F Roy C Nicaise-Roland P Palazzo E. High anti-CCP antibody titres predict good response to rituximab in patients with active rheumatoid arthritis. Joint Bone Spine. (2014) 81:416–20. doi: 10.1016/j.jbspin.2014.06.001, PMID: 24998790

[45] Courvoisier DS Chatzidionysiou K Mongin D Lauper K Mariette X Morel J. The impact of seropositivity on the effectiveness of biologic anti-rheumatic agents: results from a collaboration of 16 registries. Rheumatology. (2021) 60:820–8. doi: 10.1093/rheumatology/keaa393, PMID: 32810263

[46] Julià A López-Lasanta M Blanco F Gómez A Haro I Mas AJ. Interactions between rheumatoid arthritis antibodies are associated with the response to anti-tumor necrosis factor therapy. BMC Musculoskelet Disord. (2021) 22:372. doi: 10.1186/s12891-021-04248-y, PMID: 33882889 PMC8061050

[47] Sanmartí R Corominas H. Upadacitinib for patients with rheumatoid arthritis: a comprehensive review. J Clin Med. (2023) 12:1734. doi: 10.3390/jcm12051734, PMID: 36902522 PMC10002765

[48] Traves PG Murray B Campigotto F Galien R Meng A Di Paolo JA. JAK selectivity and the implications for clinical inhibition of pharmacodynamic cytokine signalling by filgotinib, upadacitinib, tofacitinib and baricitinib. Ann Rheum Dis. (2021) 80:865–75. doi: 10.1136/annrheumdis-2020-219012, PMID: 33741556 PMC8237188

[49] Fleischmann R Kremer J Cush J Schulze-Koops H Connell CA Bradley JD. ORAL-SOLO investigators. Placebo-controlled trial of tofacitinib monotherapy in rheumatoid arthritis. N Engl J Med. (2012) 367:495–507. doi: 10.1056/NEJMoa1109071, PMID: 22873530

[50] Lee EB Fleischmann R Hall S Wilkinson B Bradley JD Gruben D. ORAL-START investigators. Tofacitinib versus methotrexate in rheumatoid arthritis. N Engl J Med. (2014) 370:2377–86. doi: 10.1056/NEJMoa1310476 24941177

[51] Fleischmann R Schiff M van der Heijde D Ramos-Remus C Spindler A Stanislav M. RA-BEGIN investigators. Baricitinib, methotrexate, or combination in patients with rheumatoid arthritis and no or limited prior disease-modifying Antirheumatic drug treatment. Arthritis Rheumatol. (2017) 69:506–17. doi: 10.1002/art.39953, PMID: 27723271 PMC5347954

[52] Smolen JS Pangan AL Emery P Rigby W Tanaka Y Vargas JI. SELECT-MONOTHERAPY investigators. Upadacitinib as monotherapy in patients with active rheumatoid arthritis and inadequate response to methotrexate (SELECT-MONOTHERAPY): a randomised, placebo-controlled, double-blind phase 3 study. Lancet. (2019) 393:2303–11. doi: 10.1016/S0140-6736(19)30419-2, PMID: 31130260

[53] van Vollenhoven R Takeuchi T Pangan AL Friedman A Mohamed MF Chen S. SELECT-EARLY investigators. Efficacy and safety of Upadacitinib monotherapy in methotrexate-naive patients with moderately-to-severely active rheumatoid arthritis (SELECT-EARLY): a multicenter, multi-country, randomized, double-blind, active comparator-controlled trial. Arthritis Rheumatol. (2020) 72:1607–20. doi: 10.1002/art.41384, PMID: 32638504 PMC7589375

[54] Westhovens R Rigby WFC van der Heijde D Ching DWT Stohl W Kay J. FINCH-3 investigators. Filgotinib in combination with methotrexate or as monotherapy versus methotrexate monotherapy in patients with active rheumatoid arthritis and limited or no prior exposure to methotrexate: the phase 3, randomised controlled FINCH 3 trial. Ann Rheum Dis. (2021) 80:727–38. doi: 10.1136/annrheumdis-2020-219213, PMID: 33452004 PMC8142453

[55] Kremer J Li ZG Hall S Fleischmann R Genovese M Martin-Mola E. ORAL-SYNC investigators. Tofacitinib in combination with nonbiologic disease-modifying antirheumatic drugs in patients with active rheumatoid arthritis: a randomized trial. Ann Intern Med. (2013) 159:253–61. doi: 10.7326/0003-4819-159-4-201308200-00006, PMID: 24026258

[56] van Vollenhoven RF Fleischmann R Cohen S Lee EB García Meijide JA Wagner S. ORAL-STANDARD investigators. Tofacitinib or adalimumab versus placebo in rheumatoid arthritis. N Engl J Med. (2012) 367:508–19. doi: 10.1056/NEJMoa1112072, PMID: 22873531

[57] van der Heijde D Tanaka Y Fleischmann R Keystone E Kremer J Zerbini C. ORAL-SCAN investigators. Tofacitinib (CP-690,550) in patients with rheumatoid arthritis receiving methotrexate: twelve-month data from a twenty-four-month phase III randomized radiographic study. Arthritis Rheum. (2013) 65:559–70. doi: 10.1002/art.37816, PMID: 23348607

[58] Burmester GR Blanco R Charles-Schoeman C Wollenhaupt J Zerbini C Benda B. ORAL-STEP investigators. Tofacitinib (CP-690,550) in combination with methotrexate in patients with active rheumatoid arthritis with an inadequate response to tumour necrosis factor inhibitors: a randomised phase 3 trial. Lancet. (2013) 381:451–60. doi: 10.1016/S0140-6736(12)61424-X, PMID: 23294500

[59] Taylor PC Keystone EC van der Heijde D Weinblatt ME Del Carmen ML Reyes Gonzaga J. RA-BEAM investigators. Baricitinib versus placebo or Adalimumab in rheumatoid arthritis. N Engl J Med. (2017) 376:652–62. doi: 10.1056/NEJMoa1608345 28199814

[60] Dougados M van der Heijde D Chen YC Greenwald M Drescher E Liu J. RA-BUILD investigators. Baricitinib in patients with inadequate response or intolerance to conventional synthetic DMARDs: results from the RA-BUILD study. Ann Rheum Dis. (2017) 76:88–95. doi: 10.1136/annrheumdis-2016-210094, PMID: 27689735 PMC5264214

[61] Genovese MC Kremer J Zamani O Ludivico C Krogulec M Xie L. RA-BEACON investigators. Baricitinib in patients with refractory rheumatoid arthritis. N Engl J Med. (2016) 374:1243–52. doi: 10.1056/NEJMoa1507247, PMID: 27028914

[62] Burmester GR Kremer JM Van den Bosch F Kivitz A Bessette L Li Y. SELECT-NEXT investigators. Safety and efficacy of upadacitinib in patients with rheumatoid arthritis and inadequate response to conventional synthetic disease-modifying anti-rheumatic drugs (SELECT-NEXT): a randomised, double-blind, placebo-controlled phase 3 trial. Lancet. (2018) 391:2503–12. doi: 10.1016/S0140-6736(18)31115-2, PMID: 29908669

[63] Fleischmann R Pangan AL Song IH Mysler E Bessette L Peterfy C. SELECT-COMPARE investigators. Upadacitinib versus placebo or Adalimumab in patients with rheumatoid arthritis and an inadequate response to methotrexate: results of a phase III, double-blind, randomized controlled trial. Arthritis Rheumatol. (2019) 71:1788–800. doi: 10.1002/art.41032 31287230

[64] Genovese MC Fleischmann R Combe B Hall S Rubbert-Roth A Zhang Y. SELECT-BEYOND investigators. Safety and efficacy of upadacitinib in patients with active rheumatoid arthritis refractory to biologic disease-modifying anti-rheumatic drugs (SELECT-BEYOND): a double-blind, randomised controlled phase 3 trial. Lancet. (2018) 391:2513–24. doi: 10.1016/S0140-6736(18)31116-4, PMID: 29908670

[65] Combe B Kivitz A Tanaka Y van der Heijde D Simon JA Baraf HSB. FINCH-1 investigators. Filgotinib versus placebo or adalimumab in patients with rheumatoid arthritis and inadequate response to methotrexate: a phase III randomised clinical trial. Ann Rheum Dis. (2021) 80:848–58. doi: 10.1136/annrheumdis-2020-219214, PMID: 33504485 PMC8237199

[66] Genovese MC Kalunian K Gottenberg JE Mozaffarian N Bartok B Matzkies F. FINCH-2 investigators. Effect of Filgotinib vs placebo on clinical response in patients with moderate to severe rheumatoid arthritis refractory to disease-modifying Antirheumatic drug therapy: the FINCH 2 randomized clinical trial. JAMA. (2019) 322:315–25. doi: 10.1001/jama.2019.9055, PMID: 31334793 PMC6652745

[67] Retuerto M Trujillo E Valero C Fernandez-Espartero C Soleto CY Garcia-Valle A. Efficacy and safety of switching Jak inhibitors in rheumatoid arthritis: an observational study. Clin Exp Rheumatol. (2021) 39:453–5. doi: 10.55563/clinexprheumatol/cbanza, PMID: 33938793

